# Cuticular Hydrocarbon Composition of Adhesive Secretions from Functionally Different Attachment Pads of the Stick Insect *Medauroidea extradentata* (Phasmatodea)

**DOI:** 10.1007/s10886-026-01713-7

**Published:** 2026-04-24

**Authors:** Julian Thomas, Stanislav N. Gorb, Thomas Schmitt, Thies H. Büscher, Zsolt Kárpáti

**Affiliations:** 1 Functional Morphology and Biomechanics, Institute of Zoology, Kiel University, Am Botanischen Garten 1-9, Kiel, 24118 Germany; 2 Department of Animal Ecology and Tropical Biology Biocenter, University of Würzburg, Am Hubland, Würzburg, 97074 Germany; 3 Department of Chemical Ecology, Centre for Agricultural Research, Plant Protection Institute, HUN-REN, Fehérvári Street 132-144, Budapest, 1116 Hungary

**Keywords:** Tarsal secretion, Arolium, Euplantulae, Adhesion, Friction, GC-MS

## Abstract

During the evolution of insects, two principal types of attachment systems, hairy and smooth, evolved that enable them to attach to and move across diverse surfaces. Both rely on attachment supported by a thin film of fluid that enhances functionality. In some insects, multiple pad types occur on the same leg, each fulfilling complementary roles during attachment. Although their structural and mechanical differences are well understood, the secretion’s chemical composition and its functional implications remain largely unexplored. Stick and leaf insects (Phasmatodea) provide an ideal model to investigate these aspects, as they possess two morphologically and functionally distinct smooth attachment pads on the same tarsus: the pretarsal arolium, responsible for adhesion, and the tarsal euplantulae, specialised for friction. Using polydimethylsiloxane-coated solid-phase microextraction fibres and gas chromatography–mass spectrometry, we analysed the hydrocarbon profiles of both pad types and the body surface of *Medauroidea extradentata*. These data were compared with published profiles from other insect taxa possessing either hairy or smooth attachment systems to place our findings in a broader phylogenetic and functional context. Our analyses revealed distinct hydrocarbon compositions consistent with the specific mechanical roles of each structure. The arolium secretion was enriched with long-chain, methyl-branched hydrocarbons, likely enhancing fluidity and capillary forces, whereas the euplantulae secretion contained shorter, less methyl-branched hydrocarbons, suggesting higher viscosity, improving frictional contact. The body surface contained only *n*-alkanes, likely forming a wax-like protective layer. Comparisons across taxa revealed significant differences between smooth and hairy systems, reflecting both functional and phylogenetic influences on fluid chemistry.

**Supplementary Information:**

The online version contains supplementary material available at 10.1007/s10886-026-01713-7.

## Introduction

In order to locomote over and adhere to a wide variety of surfaces, most insects employ attachment organs located on their tarsi (Gorb 2001; Büscher and Gorb 2021). These systems generally consist of two components: the claws at the distal end of the pretarsus, used for interlocking with rough substrates, and tarsal attachment pads that enable adhesion to substrates with a smoother texture (Beutel and Gorb 2001).

During evolution, two morphologically distinct tarsal attachment pad types evolved convergently in insects: the hairy and the smooth attachment systems (Gorb 2001, 2007; Scherge and Gorb 2001). Hairy attachment systems are pads consisting of dense arrays of hair-like outgrowths that form numerous small contact points with the substrate (Federle 2006), whereas smooth attachment systems consist of soft cuticular pads that usually form a single, large contact area (Gorb 2007). Both the presence of these pads and their location on the tarsus are highly diverse and taxon-specific (Büscher and Gorb 2025).

Hairy pads convergently evolved in the holometabolous Coleoptera (Stork 1980; Liu and Liang 2016), Diptera (Langer et al. 2004; Friedemann et al. 2014), and Strepsiptera (Beutel and Gorb 2001; Pohl and Beutel 2004), as well as some phylogenetically distant Polyneoptera (Haas and Gorb 2004; Beutel and Gorb 2008; Büscher et al. 2024). Smooth pads are likewise found in both Polyneopteran lineages, such as Orthoptera (Kendall 1970), Dictyoptera (Wieland 2013; Schmitt and Betz 2017) and Phasmatodea (Büscher et al. 2018, 2019), as well as the holometabolous Hymenoptera (Federle et al. 2000, 2001).

Functionally, both systems operate similarly, as the properties of their attachment devices enable them to conform to the substrate’s macroscopic and microscopic surface profiles, increasing the real contact area and thereby enhancing intermolecular adhesion forces (Persson 2003; Persson and Scaraggi 2014; Popov 2015). Importantly, distinct attachment devices located on the same foot often act in a complementary manner, sharing the workload during locomotion. Previous studies have shown that the smooth pretarsal arolium provides adhesive forces ensuring strong attachment, while the smooth tarsal euplantulae provide friction forces for propulsion generation (Labonte and Federle 2013; Labonte et al. 2014; Büscher and Gorb 2019).

An important feature common to both systems is wet adhesion, whereby a tarsal fluid is secreted into the contact zone between the pad and the substrate, supporting multiple functions (Dirks and Federle 2011a, b; Dirks 2014). It fills microscopic surface irregularities, thereby increasing the real contact area (Drechsler and Federle 2006; Kovalev et al. 2013), and reinforces attachment via capillary and viscous forces (Stefan 1875; Ditsche and Summers 2014). The fluid also acts as a coupling agent that mitigates differences in surface chemistry (Dirks 2014; Thomas et al. 2023b). Beyond adhesion, the secretion contributes to the self-cleaning properties of the pad by binding particles (Clemente and Federle 2012; Zhang et al. 2019; Thomas et al. 2023a), reducing water loss from the pad (Lockey 1988), and facilitating chemical communication (Eltz 2006).

The tarsal secretions can fulfil these diverse functions due to their complex chemical composition (Federle et al. 2002). Chemical analyses of insect adhesive secretions across several orders, including Blattodea (Betz et al. 2016, 2018), Coleoptera (Kosaki and Yamaoka 1996; Geiselhardt et al. 2011; Gerhardt et al. 2016), Diptera (Bauchhenß 1979), Hymenoptera (Schmitt 1990; Eltz 2006), and Orthoptera (Vötsch et al. 2002; Reitz et al. 2015), reveal chemically diverse compositions typically comprising both water-soluble and lipid-soluble components. These two phases may occur as emulsions, either with lipid droplets dispersed in water (Vötsch et al. 2002) or water droplets dispersed in lipids (Federle et al. 2002; Dirks et al. 2010; Kaimaki et al. 2022). Compared to body surface secretions, the composition of tarsal secretions is similar in some groups (Kosaki and Yamaoka 1996; Eisner and Aneshansley 2000; Geiselhardt et al. 2009, 2011; Gerhardt et al. 2015), but different in others (Vötsch et al. 2002; Reitz et al. 2015; Betz et al. 2016).

The water-soluble phase often contains alcohols, saccharides, polar proteins, peptides, and carbohydrates (Vötsch et al. 2002; Reitz et al. 2015; Betz et al. 2016), while the lipid‐soluble phase is dominated by cuticular hydrocarbons (CHCs) with chain lengths ranging from C17 to C44, fatty acids, and waxes (Kosaki and Yamaoka 1996; Vötsch et al. 2002; Reitz et al. 2015). Due to the prevalence of CHCs in these secretions, they are likely to play a major role in influencing their physical properties, e.g., their fluidity, and consequently, their functions. (Menzel et al. 2019; Sprenger and Menzel, 2020). Experiments have demonstrated that changes in the hydrocarbon chain length, the number and positions of methyl-branches, and the number and positions of double-bonds influence these properties. Longer hydrocarbon chains increase the melting point through increased intermolecular interactions, while methyl-branches and *(Z)*-configurated double-bonds lead to more complex steric structures of the hydrocarbon molecules, reducing the impact of van-der-Waals interactions and decreasing the melting point, and thus leading to a higher fluidity (Blomquist and Bagnères 2010; Baumgart et al. 2022).

Because the functionality of insect attachment pads is strongly influenced by the tarsal secretions, and the properties of the tarsal secretions are determined by the CHC composition, it is plausible to assume that functionally distinct attachment pads also differ in their CHC profiles. To test this hypothesis, we analysed the CHC composition of the tarsal secretions and body surface in the stick insect *Medauroidea extradentata* (Brunner von Wattenwyl, 1907) (Phasmatodea), which possesses both the pretarsal arolium and tarsal euplantulae. This species was selected due to the extensive prior work on the attachment performance (Busshardt et al. 2012; Büscher and Gorb 2019; Burack et al. 2022; Thomas et al. 2023b), ultrastructure (Thomas et al. 2024), and mechanical properties (Thomas et al. 2025) of both attachment pad types.

Using solid-phase microextraction (SPME), we collected secretion samples from the arolium, euplantulae, and body surface of *M. extradentata* and analysed them with gas chromatography–mass spectrometry (GC–MS). To place our findings in a broader context, we further compared the CHC profiles of *M. extradentata* with the CHC profiles of already published data from other insect orders, encompassing Coleoptera (Geiselhardt et al. 2009, 2011; Gerhardt et al. 2016) with hairy attachment systems, as well as Blattodea (Gerhardt et al. 2015, 2016) and Orthoptera (Vötsch et al. 2002; Reitz et al. 2015) with smooth ones. This comparison was done for two reasons. First, as hairy and smooth attachment systems fulfil the same role through significant morphological differences, it is likely that the CHC composition and thereby, the physical properties of the tarsal secretion also differ between them. Second, this comparison enables us to make a wide taxonomic comparison of the CHC profiles of the tarsal secretion, which are similar or different compared to the profiles of the body surface secretions, and identify if the attachment pad secretions are chemically modulated. This study aims to (i) characterize and determine whether the CHC composition among the three body parts of *M. extradentata* differs or not, and if so, whether it can be correlated with their respective functions, and (ii) assess whether there are differences between the CHC profiles of insects with smooth and hairy attachment systems.

## Materials and Methods

### Insects

**Fig. 1:**
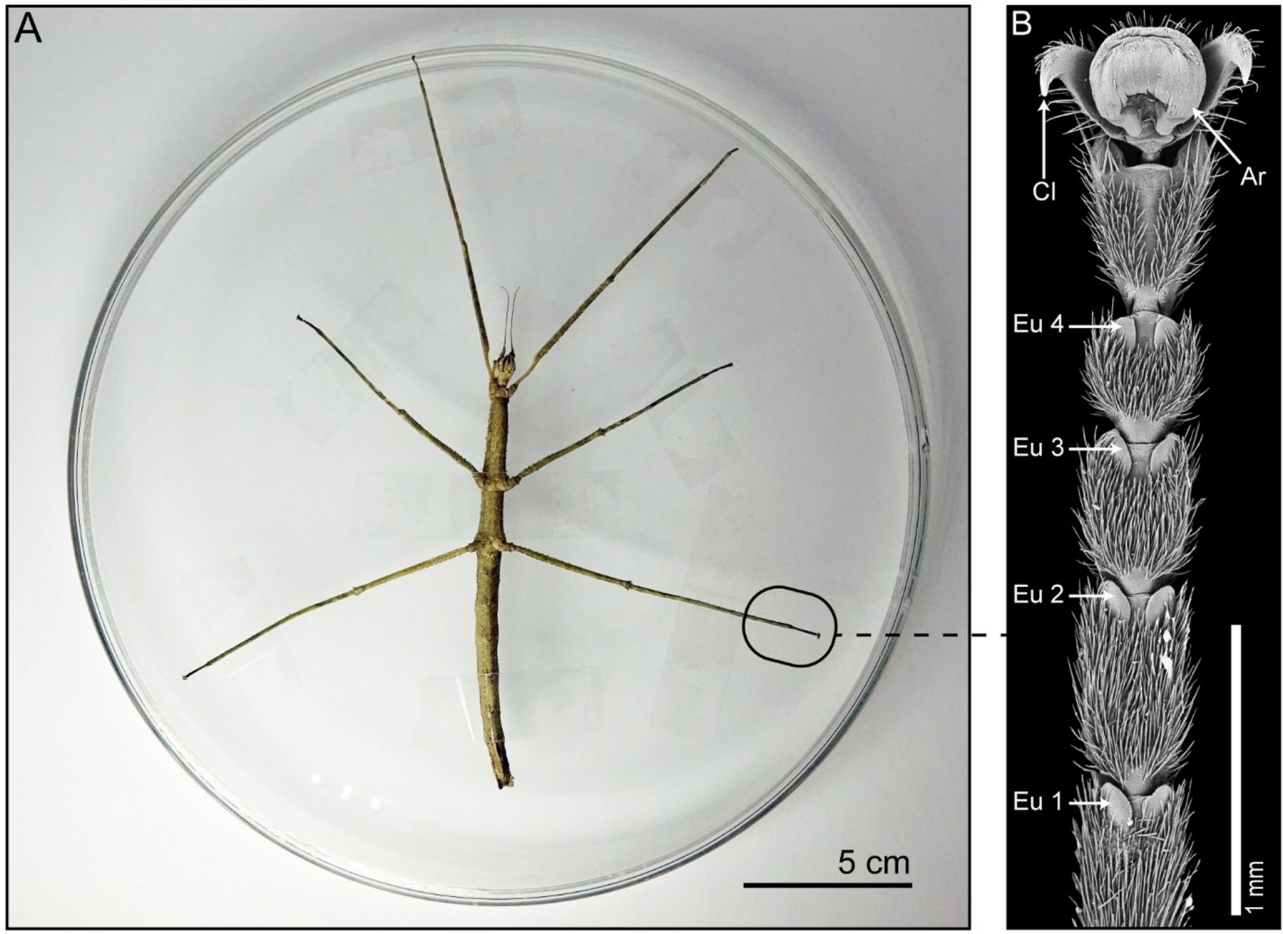
Overview of the experimental setup. **A** Female *M. extradentata* fixated on a glass plate using adhesive tape. The attachment pads on the tarsi are positioned to face up, allowing for easy access. **B** SEM image of the tarsus of *M. extradentata*. Eu 1–4 = euplantulae, Cl = claws, Ar = arolium. Figure 1B is reproduced with permission from (Thomas et al. 2023b). Copyright: The Company of Biologists

Adult females of *Medauroidea extradentata* (Phasmatodea, Clitumninae) (Fig. 1A) were selected for this study (Fig. 1B).

The insects were reared and kept in a climate chamber under a 12 L:12D photoperiod, 70% RH, 24 °C conditions, and were fed *ad libitum* with blackberry leaves. For the experiments, only females without visible damage to their attachment pads were selected, as scarring or aging affects the attachment ability and, therefore, could potentially influence the production of tarsal secretions (Grote et al. 2024).

### Extraction of the Chemical Profiles

*Medauroidea extradentata* females were anesthetized with CO_2_ for approximately 20 s and subsequently fixed on their dorsal side on a glass plate using adhesive tape, enabling access to all attachment pads on the legs and to the body surface (Fig. 1A). To remove potential contaminations, all attachment pads and the body surface were carefully cleaned with 70% ethanol and deionized water using two low-lint wipes. To allow recovery of the adhesive secretion, the animals were left undisturbed for 20 min, as previous studies on *Carausius morosus* (Phasmataodea) and *Nauphoeta cinerea* (Blattodea) demonstrated that fluid-depleted pads recover maximal footprint volumes within 15 min (Dirks and Federle 2011b). The chemical profiles were extracted using a solid phase microextraction (SPME) fibre with a 100 μm polydimethylsiloxane (PDMS) coating (Merck KGaA, Darmstadt, Germany). Before each extraction, the fibre was conditioned for 10 min at 280 °C in the injection port of a gas chromatograph. The same fibre was used for each extraction.

Samples were obtained from three body regions: the pretarsal arolium, the tarsal euplantulae, and the body surface (tibia, femur, thorax, and abdomen). Sampling was performed under a stereomicroscope to ensure that only secretions from the targeted structures were collected. For extraction, the SPME fibre was gently brushed over the respective surfaces.

Each arolium sample consisted of pooled extractions from all six arolia of a single individual. Each euplantulae sample comprised pooled extracts from all 48 euplantulae of one individual. Body surface samples were obtained from the tibia, femur, thorax, and abdomen of a single individual.

In total, six individuals were analysed for the arolium (*n* = 6), and eight individuals for both the euplantulae (*n* = 8) and the body surface (*n* = 8). Extraction of the profiles from the arolium and the euplantulae took a total of 20 min, with a five-minute break after 10 min, to allow the tarsal secretion to regenerate. Extraction of the CHC profile from the body surfaces took 20 min without a break.

### Analysis of the Hydrocarbon Profiles

The loaded SPME fibre was immediately injected for 2 min into the split/splitless injector port of an Agilent 6890 gas chromatograph coupled to an Agilent 5975 Mass Selective Detector (GC-MS, Agilent, Waldbronn, Germany) to thermally desorb the extracted compounds. The injection port of the GC was set to splitless mode at 280 °C. The GC was equipped with a DB-5 Fused Silica capillary column (30 m x 0.25 mm ID, df = 0.25 μm; J&W Scientific, Folsom, USA). Helium served as the carrier gas at a constant flow of 1 ml/min. The GC oven temperature was initially set to 50 °C, and it increased by 5 °C per minute to a maximum of 280 °C. The electron ionization mass spectra (EI-MS) were acquired at an ionization voltage of 70 eV (source temperature: 230˚C). Chromatograms and mass spectra were recorded and quantified via integrated peak areas using MassHunter software (version 10.0; Agilent). Hydrocarbons were identified by their retention indices and diagnostic ions (Carlson et al. 1998). Integrated peak areas were used to calculate the relative amount of each compound. One example chromatogram for each body region can be found in the supplementary material (Fig. 1S).

### Visualization and Statistical Analysis

To visualize the differences in the CHC profiles, we used non-metric multidimensional scaling (NMDS), based on a Bray-Curtis dissimilarity matrix. In the NMDS plot, the spatial distances between data points indicate the differences in hydrocarbon profiles between samples, while the stress value indicates how well the two-dimensional representation fits the multidimensional distances (Kruskal 1964, 1969). PERMANOVA (Permutational multivariate analysis of variance, permutations = 9999) was applied to test for differences between groups (Anderson 2001). The statistical analyses were performed in R 4.4.1 (R Core Team, 2025) using the vegan package (Oksanen et al. 2025). Data structures derived from the visualization method do not require a priori knowledge of samples representing a group. Grouping is solely based on the chemical compositions of the analysed extracts.

A Random Forest (RF) classification analysis (Breiman 2001) was conducted using the MetaboAnalyst 6.0 online platform (2025) to assess the contribution of individual hydrocarbons to the separation of the arolium, euplantulae, and body surface groups in the NMDS.

The measured hydrocarbon profiles (Table 1) were statistically analyzed using Python (Python version 3.13.4) to identify differences in the proportion of methyl-branched alkanes compared with *n*-alkanes, the average hydrocarbon chain length, and their total area as a proxy for the absolute amount of each hydrocarbon (Fig. 3). The data were tested for normal distribution (*Shapiro-Wilk Test*, *P* < 0.05) and homoscedasticity (*Levene’s Test*, *P* < 0.05) before comparison. As the data for the total area was not normally distributed, a *Kruskal-Wallis test* with a *Dunn’s post hoc* test was used for the comparison of the three samples (the significance level (alpha) was set to *P* < 0.05). The data for the average hydrocarbon chain length was normally distributed, and a *One-Way ANOVA* with a *Tukey’s post hoc* test was used for the comparison (the significance level (alpha) was set to *P* < 0.05).

**Table 1:** Relative abundances (± SD) of the identified hydrocarbons with retention indices of the arolium, the euplantulae, and the body surface of female *M. extradentata*

Compounds	Retention index	Arolium	Euplantulae	Body surface
C21	2100	0.38 ± 0.14	1.17 ± 0.17	2.12 ± 0.47
C22	2200	0.44 ± 0.11	2.29 ± 0.27	2.99 ± 0.4
C23	2300	1.33 ± 0.42	3.96 ± 0.57	3.67 ± 0.91
C24	2400	0.65 ± 0.18	2.47 ± 0.39	2.52 ± 0.77
C25	2500	2.8 ± 0.64	10.28 ± 2.39	8.3 ± 1.91
C26	2600	1.22 ± 0.26	4.27 ± 0.76	3.7 ± 1.43
C27	2700	5.91 ± 1.03	13.56 ± 2.14	12.33 ± 1.42
C28	2800	0.47 ± 0.19	0.96 ± 0.47	1.24 ± 0.37
C29	2900	7.95 ± 0.98	22.37 ± 3.32	25.45 ± 3.09
15-; 13-MeC29	2927	8.03 ± 1.47	1.01 ± 0.39	0 ± 0
7-MeC29	2937	2.11 ± 0.45	0 ± 0	0 ± 0
5-MeC29	2946	0.81 ± 0.18	0 ± 0	0 ± 0
3-MeC29	2971	2.16 ± 0.45	0 ± 0	0 ± 0
C30	3000	1.71 ± 0.28	3.8 ± 0.92	2.43 ± 0.57
15-; 14-MeC30	3027	4.78 ± 0.75	0 ± 0	0 ± 0
4-MeC30	3054	0.2 ± 0.06	0 ± 0	0 ± 0
C31	3100	7.29 ± 1.44	18.83 ± 2.23	33.41 ± 5.25
15-; 13-MeC31	3123	33.58 ± 1.4	7.26 ± 2.06	0 ± 0
7-MeC31	3135	5.41 ± 0.92	0 ± 0	0 ± 0
5-MeC31	3146	2.72 ± 0.76	0 ± 0	0 ± 0
3-MeC31	3166	4.79 ± 0.58	7.37 ± 2.31	0 ± 0
C32	3200	2.86 ± 0.29	0 ± 0	0.63 ± 0.18
14-; 13-; 12-MeC32	3222	2.28 ± 0.54	0.36 ± 0.16	0 ± 0
C33	3300	0.13 ± 0.05	0.04 ± 0.04	1.21 ± 0.33

### Comparison with Previously Published CHC Profiles

The published data were selected based on the following requirements: (i) The hydrocarbon profiles of insects must have been identified. (ii) The data had to include the hydrocarbon profiles of the adhesive secretion and the CHC profile of the body surface. iii) The relative abundance of each hydrocarbon, along with its retention index, must have been reported. All substances not classified as hydrocarbons were removed from the comparison, as we focus on hydrocarbons only. The following publications were selected based on these criteria: (Vötsch et al. 2002; Geiselhardt et al. 2009, 2011; Gerhardt et al. 2015, 2016; Reitz et al. 2015). The published data contains hydrocarbons in which the exact position of their internal methyl-branches in the molecule was not identified; such CHCs are marked as i-Me. To visualize the differences between the CHC profiles and to assess the contribution of individual hydrocarbons to their separation, NMDS and RF analyses were used (as described above). The table containing all the hydrocarbon profiles from the selected studies can be found in the supplementary information (Table S1).

## Results

### Hydrocarbon Profiles of Different Body Parts of *M. extradentata*

The hydrocarbon profiles of the arolium, euplantulae, and body surface of *M. extradentata* exhibited significant compositional differences (Table 1). Two major classes of hydrocarbons, *n*-alkanes and monomethyl-branched alkanes, were identified, and their relative amounts were calculated, with chain lengths ranging from C21 to C33. The presence and relative abundance of these compounds varied across the three body regions (Table 1).

**Fig. 2:**
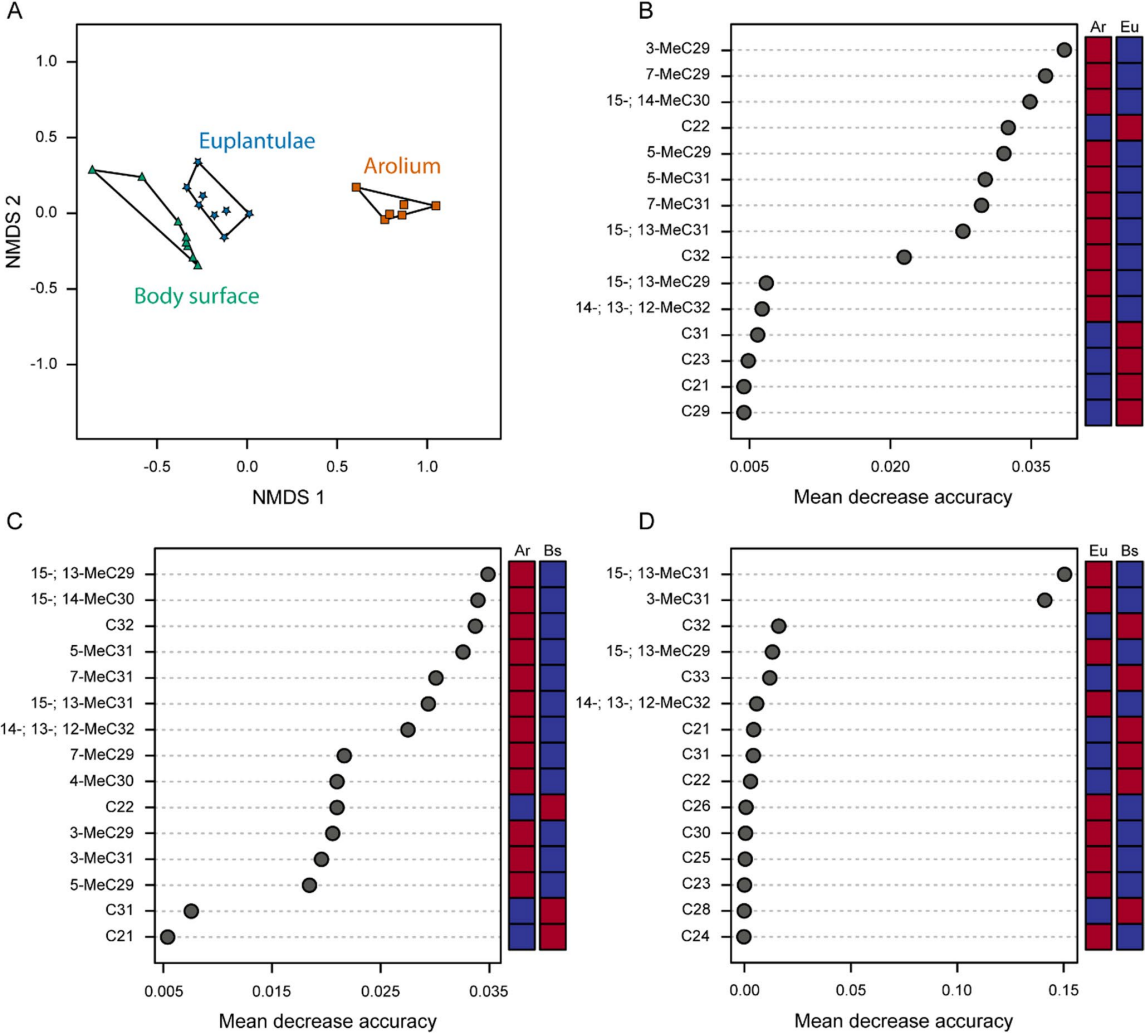
NMDS and Random Forest analyses of hydrocarbon profiles from the arolium, euplantulae, and body surface of *M. extradentata*. **A** NMDS of the three hydrocarbon profiles. Each point represents a single sample. Green triangles = CHC profile of the body surface (*n* = 8), blue stars = hydrocarbon profile from the euplantulae (*n* = 8), and orange squares = hydrocarbon profile from the arolium (*n* = 6). Connected samples are of the same secretion type. All three groups form significantly distinct clusters (PERMANOVA, *p* < 0.05 for all comparisons). (B – D) Random Forest analysis of the hydrocarbon profiles of the: (**B**) arolium (Ar) and euplantulae (Eu); (**C**) arolium (Ar) and body surface (Bs); (**D**) euplantulae (Eu) and body surface (Bs). On the y-axis are the 15 most influential hydrocarbons ranked by their mean decrease accuracy (mda). A higher mda value has a larger contribution to group separation. The squares on the right display the abundance of the corresponding hydrocarbons, with blue indicating low and red high abundance

The NMDS visualizes the differences between the hydrocarbon profiles of the three body parts of *M. extradentata* (arolium, euplantulae, and body) (Fig. 2). It demonstrates that the hydrocarbon profiles of each body part form their own cluster, which differ significantly from the other clusters (PERMANOVA, *df* = 2, *F* = 21.031 arolium vs. body surface, *P* = 0.003; arolium vs. euplantulae, *P* = 0.003; euplantulae vs. body surface, *P* = 0.045) (Fig. 2A). Random Forest classification identified key hydrocarbons responsible for the separation of the groups. For the comparison between arolium and euplantulae profiles, the most discriminative compounds were: 3-MeC29 (0.038 mean decrease accuracy (m.d.a.)), 7-MeC29 (0.036 m.d.a.), 15-; 14-MeC30 (0.034 m.d.a.) (all enriched in the arolium profile), and C22 (0.032 m.d.a.) (enriched in the euplantulae profile) (Fig. 2B). In the comparison between arolium and body surface profiles, 15-; 13-MeC29 (0.034 m.d.a.), 15-; 14-MeC30 (0.033 m.d.a.), C32 (0.033 m.d.a.), and 5-MeC31 (0.032 m.d.a.) were the most discriminative compounds, and all of them were more abundant in the arolium profile (Fig. 2C). The Random Forest analysis for the comparison between euplantulae and the body surface profiles showed the lowest m.d.a. values of all comparisons, with the compounds 15-; 13-MeC31 (0.15 m.d.a.) and 3-MeC31 (0.14 m.d.a.) being the most discriminative compounds, and they are more abundant in the euplantulae profile (Fig. 2D).

**Fig. 3:**
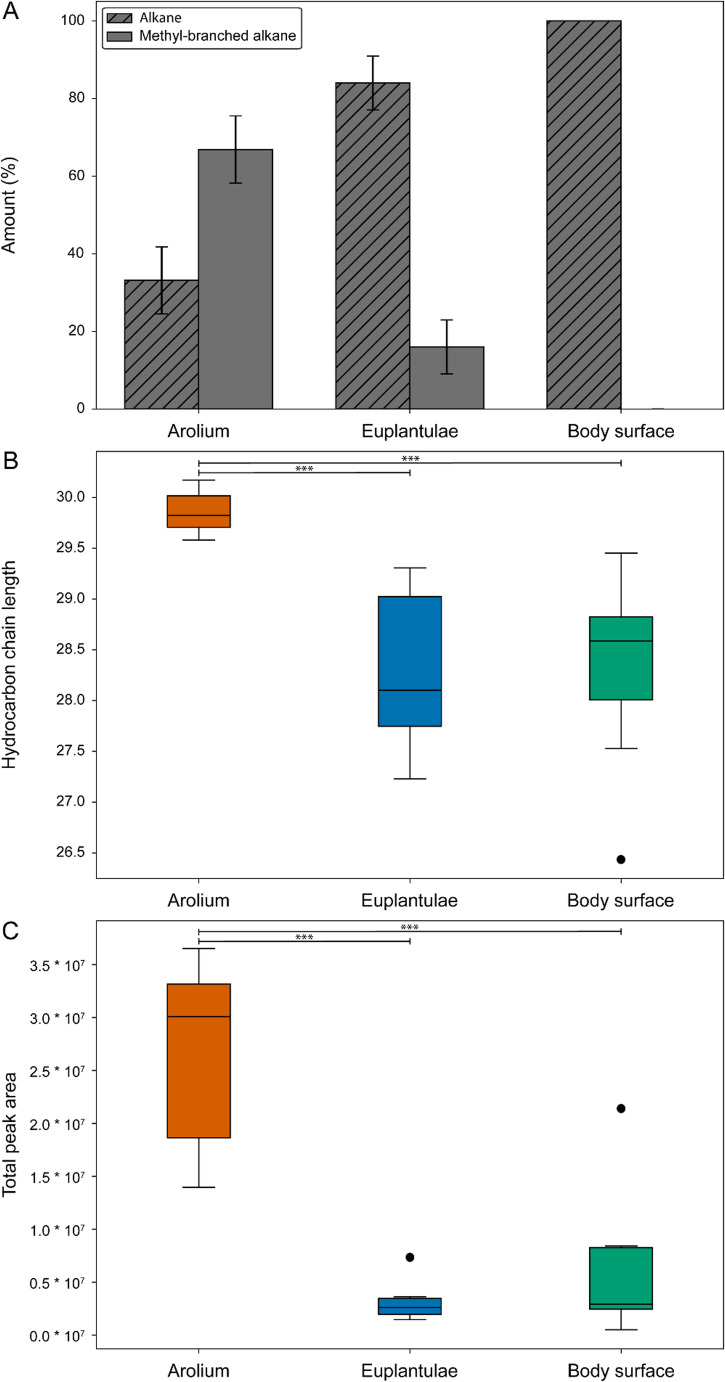
Analysis of hydrocarbon profiles of the arolium, euplantulae, and body surface. **A** Relative amounts of *n*-alkanes (grey with lines) and methyl-branched alkanes (grey). **B** Hydrocarbon chain length of the three groups, and (**C**) total peak areas of the hydrocarbon profiles of each group. Boxes represent the 25th to 75th percentiles, whiskers extend to the 10th and 90th percentiles, and the line within each box indicates the median, *** = *P* < 0.001

Further differences among the three profiles were investigated by comparing the proportions of methyl-branched alkanes and *n*-alkanes, average chain length, and peak area as a proxy for the absolute amount of each compound (Fig. 3). The arolium profile exhibited the highest proportion of monomethyl-branched hydrocarbons (66.86 ± 8.65%), with the remainder being *n*-alkanes (33.13 ± 8.65%). The euplantulae profile showed a lower proportion of monomethyl-branched hydrocarbons (16.01 ± 6.94%) and a predominance of *n*-alkanes (83.98 ± 6.94%). In contrast, the CHC profile of the body surface was composed exclusively of *n*-alkanes (100%) (Fig. 3A).

The average hydrocarbon chain length was significantly longer in the arolium (29.85 ± 0.22) compared to both the euplantulae (28.28 ± 0.77) and the body surface (28.33 ± 0.97), but showed no differences between the euplantulae and body surface (*One-Way ANOVA*,* df* = 19, *F* = 8.97, *P* = 0.002 with *Tukey’s post hoc* test, arolium vs. euplantulae *P* = 0.0035; arolium vs. body *P* = 0.0073; euplantulae vs. body *P* = 1) (Fig. 3B).

The peak area as a proxy for the absolute amount of all hydrocarbons measured, was the largest in the arolium profile (2.66 × 10⁷ ± 0.96 × 10⁷), significantly exceeding the peak area of the profile of the euplantulae (0.31 × 10⁷ ± 0.18 × 10⁷) and of the body (0.60 × 10⁷ ± 0.68 × 10⁷), with the latter two not statistically different from each other (Kruskal-Wallis test, *H* = 11.65, *P* = 0.003 with Dunn’s *post hoc* test, arolium vs. euplantulae *P* = 0.0043; arolium vs. body *P* = 0.014; euplantulae vs. body *P* = 1) (Fig. 3C).

### Hydrocarbon Profiles of Tarsal Secretions and The Body Surface of Different Insect Species

**Fig. 4:**
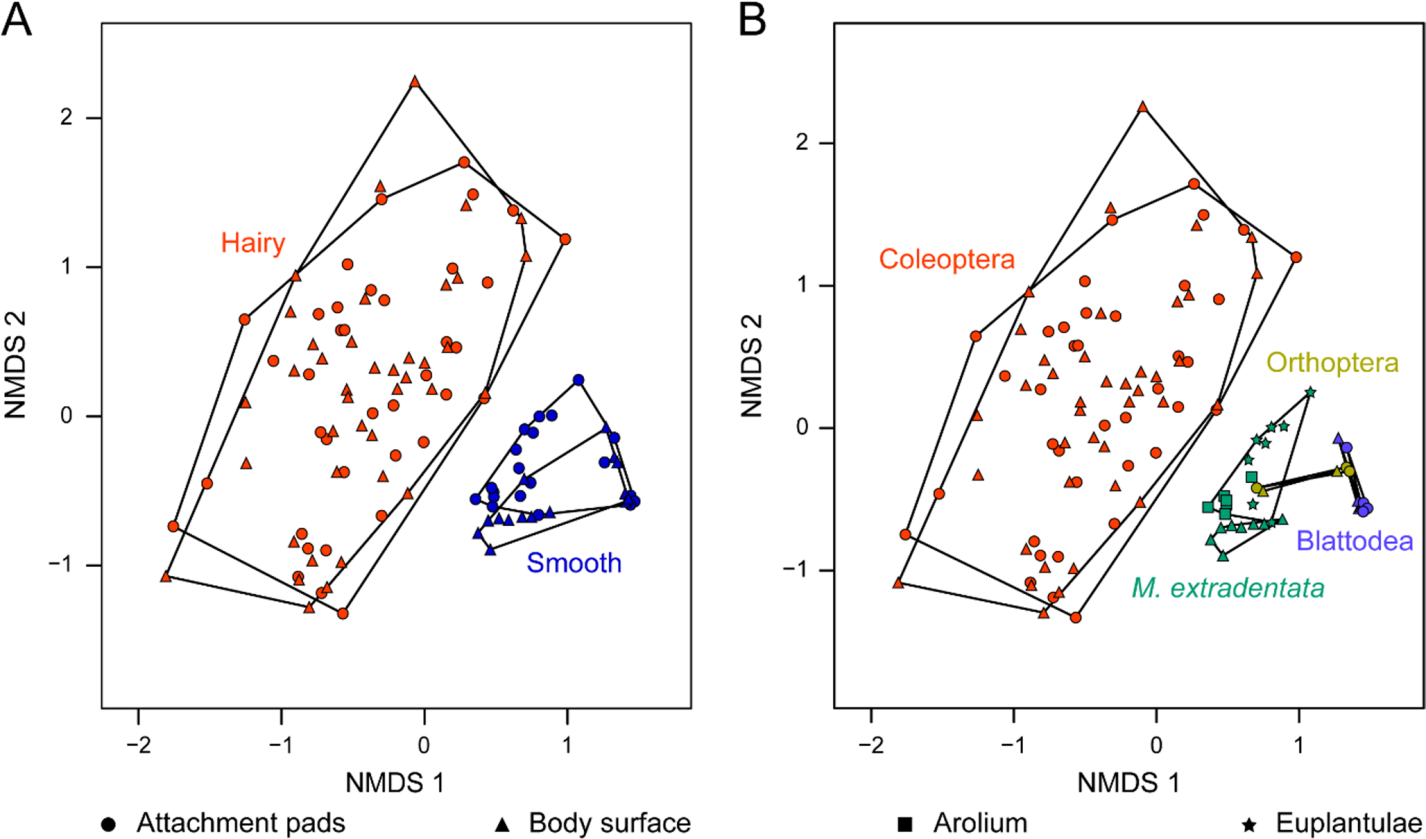
NMDS of hydrocarbon profiles of different insect species grouped by attachment system type (A) or by order (B). **A** NMDS of the different insect species based on the type of attachment system. Red circles = hydrocarbon profiles of the hairy attachment pads (*n* = 38), red triangles = CHC profiles of the hairy body surface (*n* = 40), blue circles = hydrocarbon profiles of the smooth attachment pads (*n* = 20), blue triangles = CHC profiles of the smooth body surface (*n* = 15). **B** NMDS of the different insect species based on their order. Red circles = hydrocarbon profiles of the attachment pads of Coleoptera (*n* = 38), red triangle = CHC profiles of the body surface of Coleoptera (*n* = 40), green squares = hydrocarbon profiles of the arolium of *M. extradentata* (Phasmatodea) (*n* = 6), green stars = hydrocarbon profiles of the euplantulae of *M. extradentata* (Phasmatodea) (*n* = 8), green triangle = CHC profiles of the body surface of *M. extradentata* (Phasmatodea) (*n* = 8), dark yellow circle = hydrocarbon profiles of the attachment pads of Orthopera (*n* = 3), dark yellow triangles = CHC profiles of the body surface of Orthoptera (*n* = 2), purple circles = hydrocarbon profiles of the attachment pads of Blattodea (*n* = 4), purple triangles = CHC profiles of the body surface of Blattodea (*n* = 4). Each data point represents a single sample

The NMDS generated from the hydrocarbon profiles of *M. extradentata* (arolium, euplantulae and body surface), integrated with previously published hydrocarbon profile data of adhesive organs and body surfaces of various insect species revealed a clear separation in the attachment pad and the body surface hydrocarbon profiles between different insect taxa with hairy and smooth attachment systems (PERMANOVA, hairy pad secretion vs. smooth surface CHC profile *P* = 0.0015, hairy pad secretion vs. smooth pad secretion *P* = 0.0015, hairy surface CHC profile vs. smooth surface CHC profile *P* = 0.0015, hairy surface CHC profile vs. smooth pad secretion *P* = 0.0015) (Fig. 4A). In insects with hairy systems, the CHC profiles of attachment pads and the body surface were not statistically different from each other (PERMANOVA, hairy pad secretion vs. hairy surface profile, *P* = 1). In contrast, the hydrocarbon profiles of the pad secretions and the body surface of species with smooth attachment systems (including species from Phasmatodea, Blattodea, and Orthoptera) were found to differ significantly although the cluster slightly overlap in the NMDS (PERMANOVA, smooth pad secretion vs. smooth surface CHC profile, *P* = 0.012) (Figure 4A). Random Forest analysis for the comparisons of the body surface (Figure S2) and attachment pad secretions (Figure S3) between insects possessing hairy and smooth attachment systems are visualized in supplementary information.

To compare the phylogenetic background of the hydrocarbon profiles, they were visualized across different insect orders in an NMDS with the same data as in Fig. 4A. PERMANOVA indicated significant differences between the hydrocarbon profiles of Coleoptera and all other orders (PERMANOVA, *P* < 0.05 for all comparisons). Within each insect order, the hydrocarbon profiles of attachment pads and the body surface overlapped in the NMDS and showed no differences (PERMANOVA, Coleoptera attachment pad vs. Coleoptera body, *P* = 1; Blattodea attachment pad vs. Blattodea body, *P* = 0.16; Orthoptera attachment pad vs. Orthoptera body, *P* = 0.93). The only exception was *M. extradentata* (Phasmatodea), where the hydrocarbon profile of the pretarsal arolium significantly differed from the tarsal euplantulae and the body surface (PERMANOVA, *P* > 0.05 for both comparisons) (Fig. 4B). Random Forrest analysis for the comparison of the body surface and attachment pad secretion within Blattodea (Figure S4) is included in supplementary information. Comparison within Orthoptera was not possible due to the low number of representatives.

## Discussion

### Differences in the CHC Profile Between the Secretions of *M. extradentata*

This study provides the first detailed comparative analysis of the hydrocarbon composition of the secretions of the pretarsal arolium, the tarsal euplantulae, and the body surface of *M. extradentata* females (Figs. 2 and 3). Our results reveal pronounced qualitative and quantitative differences among these profiles, indicating chemical and, consequently, functional adaptation of the secretions to the specific mechanical roles of each body region.

The hydrocarbon fraction of all three secretions consists exclusively of saturated hydrocarbons. While both the arolium and euplantulae secretions contain *n*-alkanes and methyl-branched alkanes, the arolium shows a markedly higher proportion of methyl-branched compounds. In contrast, the secretion from the body surface contains only *n*-alkanes (Table 1; Figs. 2 and 3). Such differences, where an entire class of hydrocarbons is present in one structure but absent in another, and where the two attachment pads located on the same leg possess different CHC profiles, have not been reported previously. To our knowledge, previous studies analysing tarsal secretions did not explicitly distinguish between different attachment pad types on the same tarsus (Vötsch et al. 2002; Geiselhardt et al. 2009, 2011; Gerhardt et al. 2015, 2016; Reitz et al. 2015; Huthmacher and Menzel 2026).

Multiple mechanisms could potentially account for this compositional difference. A distinct novel biosynthetic pathway could be activated to produce methyl-branched hydrocarbons in the glandular tissue of the attachment pads (Thomas et al. 2024), or the lipophorin transport system selectively carries these substances to the tarsal structures of *M. extradentata* (Haruhito and Haruo 1982; Blomquist and Bagnères 2010). These possibilities remain to be tested experimentally. The explicit presence of methyl-branched hydrocarbons and different average chain lengths in the attachment pad secretions suggests that they possess a specific effect on the function of their respective secretion. Notably, the CHC profiles of *M. extradentata* are among the simplest compositions described for insect secretions so far, e.g. (Vötsch et al. 2002; Geiselhardt et al. 2011; Reitz et al. 2015; Gerhardt et al. 2016). This chemical simplicity likely reflects a basic structural framework that can be locally modified to meet the mechanical demands of different functions of attachment pads.

The arolium secretion is characterized by a significant enrichment of methyl-branched alkanes and longer average chain lengths compared to the secretions of the euplantulae and body surface (Figs. 2 and 3A and B). The combination of long-chain hydrocarbons, which increase the melting point through enhanced van der Waals interactions, and a high proportion of methyl-branched alkanes, which disrupt intermolecular packing due to their non-linear structure (Poger et al. 2014), likely results in a secretion whose fluidity is maintained across ambient temperatures. (Gibbs 1995; Blomquist and Bagnères 2010). This potential fluidity would increase surface wettability, promoting a larger contact area, filling surface roughness, and, consequently, higher adhesion forces. Additionally, the secretion could facilitate capillary forces, thereby enhancing attachment further (Langer et al. 2004; Drechsler and Federle 2006; Persson 2007; Ditsche and Summers 2014). The higher fluidity of the arolium secretion may also be reflected in its significantly higher total peak area compared with the euplantulae and body surface (Fig. 3C), as a more fluid secretion would possess a higher absorption rate. However, this comparison must be interpreted cautiously. Previous studies and our own observations show that the arolium is significantly softer than the other two structures (Thomas et al. 2025), resulting in a larger contact area with the SPME fibre. This increased contact area could also enhance compound transfer and thus contribute to the measured higher peak areas.

In comparison, the euplantulae secretion contains shorter-chained and less methyl-branched hydrocarbons (Figs. 2 and 3A and B), likely leading to a higher melting point and higher viscosity at ambient temperatures (Gibbs 1995; Blomquist and Bagnères 2010). This potentially more viscous secretion would enhance resistance to shear forces during locomotion by reducing pad deformation and promoting contact stability (Gorb et al. 2002; Busshardt et al. 2012; Labonte and Federle 2013). It may also reduce the evaporation rate of the secretion, maintaining a stable fluid film during prolonged surface contact (Lockey 1988; Vötsch et al. 2002; Dirks and Federle 2011a; Amador et al. 2024). The CHC profile of the body surface secretion only consists of short-chained *n*-alkanes, without any methyl-branched hydrocarbons (Table 1; Figs. 2 and 3A and B). This could allow a more dense molecular packing and a high melting point, resulting in a solid, wax-like layer that functions as a hydrophobic barrier against water loss and microbial colonization (Gibbs 1995; Blomquist and Bagnères 2010; Menzel et al. 2019).

Taken together, our findings support the hypothesis that the CHC composition differs between the three sample types of *M. extradentata* according to their functional specializations. The body surface’s CHC profile appears to represent a baseline hydrocarbon profile composed solely of *n*-alkanes, while the two tarsal pads exhibit modifications tailored to their mechanical roles. In the case of the euplantulae, only minor modifications to the hydrocarbon profile appear to be necessary for adaptation to the friction enhancement. Their secretion retains much of the structure of the body surface CHC profile, being modified only by minor amounts of methyl-branched alkanes (Gibbs 1995). In contrast, the arolium profile exhibits substantial chemical deviation, particularly through an elevated number and amounts of methyl-branched alkanes and longer carbon chains. These modifications likely reflect the demands of adhesion, where increased wettability and higher capillary forces are essential. Differences between the hydrocarbon profile of the tarsal attachment pads and the CHC profile of the body surface have also been shown by Gerhardt et al. (2016). However, they only found differences in the amount and not the complete absence of methyl-branched hydrocarbons in the CHC profile of the body surface (Gerhardt et al. 2016).

It should be noted that our analyses represent only one aspect of the complex chemistry of tarsal secretions and are based on relative peak areas. Consequently, the data reflect only one class of compounds and the relative composition of hydrocarbons rather than their absolute quantities. Differences in the total volume of secretion released by different pad types could influence the rheological properties of the fluid and its contribution to attachment performance. Such effects cannot be assessed from relative compositional data alone. Future studies analysing additional substance classes and incorporating quantitative measurements of secretion volume will therefore be necessary to obtain a more complete understanding of tarsal secretion chemistry and functionality.

### Comparative Analysis Across Attachment Systems and Insect Orders

To address whether the hydrocarbon profiles differ between insects with smooth and hairy attachment systems, and how the CHC composition of the body surface is related to that of the attachment pads, we compared our results with published data on tarsal secretions and body surface hydrocarbons across various insect taxa.

Interpretation of hydrocarbon profiles across the selected studies is complicated by variation in sampling methods and the body regions analysed (Vötsch et al. 2002; Geiselhardt et al. 2009, 2011; Gerhardt et al. 2015, 2016; Reitz et al. 2015). Previous studies used different collection methods, including SPME-, glass- or PDMS-fibres, solvent extraction, and washes of entire footprints. In addition, several studies do not clearly specify whether the analysed tarsal CHC profiles included the profiles of individual attachment pad types (e.g. arolium or euplantulae in smooth attachment systems) or pooled secretions from multiple structures. Such methodological differences make it difficult to disentangle potential variation in hydrocarbon composition, particularly between different pad types, and may obscure fine-scale patterns in secretion chemistry. Furthermore, differences in pad size and total secretion volume between species may also influence the detectability and functional balance of CHC profiles.

An exception is the study of Reitz et al. (2015), which analysed CHC profiles from different tarsal structures of *Schistocerca gregaria*, including attachment pads and tibiae, using both solvent extraction and SPME (Reitz et al. 2015). While significant differences were found between attachment pads and tibiae, no differences were detected between solvent extracts containing pooled pad secretions from the arolia and euplantulae and SPME samples obtained from the euplantulae alone. This suggests that the CHC profiles of the arolium and euplantulae in *S. gregaria* may be broadly similar and further indicates that differences in sampling method may not necessarily account for all variation observed between studies.

Nevertheless, this comparative analysis revealed clear distinctions between the two attachment system types, with insects possessing smooth systems exhibiting significantly different hydrocarbon profiles of their attachment pads and their body surfaces from those with hairy systems (Fig. 4A). Random Forest analyses identified the long-chain hydrocarbons C29, C30, and C31 as the main contributors to this separation, all of which were more abundant in smooth systems (Figures S2 and S3). As all the profiles from the hairy systems originate from Coleoptera species and the profiles from the smooth systems from specimens of the orders Blattodea, Orthoptera, and Phasmatodea, the differences could be a result of either their phylogenetic divergence (Misof et al. 2014) or due to different requirements of the secretion during contact formation.

The phylogenetic explanation is plausible given that Coleoptera are distantly related to the remaining polyneopteran groups (Misof et al. 2014) hence, differences may be linked to lineage-specific hydrocarbon evolution.

It is also likely that the required physical properties of the secretions are different between the hairy and smooth attachment systems due to the different contact formation (Büscher and Gorb 2023). The different physical mechanisms both attachment pads use to generate attachment forces might pose different requirements for voluminal and rheological properties of the secretions. While the hairy systems generate multiple but very small individual contacts, in smooth systems, one large contact zone is generated by the whole attachment pad (Gorb 2001; Bullock et al. 2008). This likely necessitates different physico-chemical properties of the secretion to sustain optimal viscosity and film thickness and thus would influence the hydrocarbon composition. An indication of these different properties is the presence of the three longer CHCs (C29, C30, and C31) in the smooth attachment systems, which would result in potentially higher viscous secretion (Figures S2 and S3). Supporting this further, rheological measurements exist for hairy pad secretions (Peisker et al. 2014) but are lacking for smooth systems, possibly because their secretions are more viscous and difficult to sample in sufficient quantities and measure using methods established for hairy pads (pers. observation).

To evaluate how the CHC composition of the body surface is related to that of the attachment pads, we analyzed the CHC profiles within both attachment system types (smooth and hairy) and across insect orders (Coleoptera, Blattodea, Orthoptera, and Phasmatodea) using NMDS and RF analyses (Figs. 4B, S4). In nearly all examined taxa and attachment system types, the CHC profiles of the secretions of the body surface and the attachment pads were statistically indistinguishable, indicating largely uniform CHC compositions between them (Figs. 4B, S4). This pattern suggests that, in both smooth and hairy systems, the overall CHC composition remains broadly consistent across different body regions, although minor adjustments in the relative abundance of specific compounds could locally modulate the fluidity of the pad secretion to optimize performance (see also Vötsch et al. 2002). The only clear exception to this trend was observed in *M. extradentata*, where the CHC profile of the arolium secretion differed significantly from that of both the body surface and the euplantulae. This distinct clustering highlights the functional differentiation between the two types of smooth attachment pads: while the euplantulae are primarily involved in generating friction, the arolium is specialized for adhesion, which likely requires a secretion of different chemical and rheological properties.

In summary, we show clear pad-specific differences in their CHC profiles within *M. extradentata*, indicating that the chemistry of the secretions is modified to support the functional specialization of their body parts. Our comparative analysis indicates that hydrocarbon compositions of insect tarsal secretions are generally conserved across body regions and attachment systems. Distinct chemical divergence, such as between the friction-generating euplantulae and adhesion-generating arolium of *M. extradentata*, appears to arise only in response to clear functional differentiation. However, our conclusions are based on the relative peak areas of the CHC profiles and the functional properties of the attachment pads and not on the absolute quantities of the secretion nor their rheological attributes. Consequently, the proposed functional implications of the fluid compositions require further experimental investigation.

## Conclusion

This study demonstrates that the hydrocarbon profile of insect secretions is functionally specialised to support the mechanical demands of insect attachment pads. In *M. extradentata*, the secretion of the pretarsal arolium is chemically distinct, enriched in long-chain, methyl-branched hydrocarbons, potentially tuning the viscosity of the fluid for adhesion. In contrast, the hydrocarbon profile of the secretion of the tarsal euplantulae is more similar to the body CHC profile, with minor amounts of methyl-branched alkanes. This suggests more viscous properties that might support friction performance. The body secretion, composed exclusively of linear *n*-alkanes, likely forms a wax-like barrier to protect the body against evaporation.

These findings suggest that attachment secretions are derived from the body fluid and selectively modified according to pad function. The degree of chemical modifications correlates with the pad’s mechanical role, with more modifications present in structures responsible for generating adhesion (e.g., the arolium).

Our comparative analysis across insect taxa highlights that in hairy attachment systems, CHC compositions remain consistent between body and pad secretions, indicating minimal modification. In contrast, smooth attachment systems, particularly the pretarsal arolium, show more pronounced modifications, underlining the role of functional demand in changing the properties of the secretion. Further research is required to disentangle phylogenetic and functional backgrounds of CHC composition and corroborate the hypothetical compositional difference between secretions of hairy and smooth attachment systems across insects. It is also important to note that CHCs represent only one component of the complex secretion, and a comprehensive understanding of its physico-chemical properties and functional role requires analysing the other constituents as well.

## Supplementary Information

Below is the link to the electronic supplementary material.

Supplementary Material 1

## Data Availability

All relevant data can be found within the article and its supplementary information.
