# Supplementary material for: Cuticular Hydrocarbon Composition of Adhesive Secretions from Functionally Different Attachment Pads of the Stick Insect Medauroidea extradentata (Phasmatodea)
Source: J Chem Ecol. 2026 Apr 24;52(3):39. doi: 10.1007/s10886-026-01713-7 (PMC13109221; doi:10.1007/s10886-026-01713-7)
Supplement: Supplementary file 1 — Supplementary Material 1 [file 10886_2026_1713_MOESM1_ESM.pdf]

SUPPLEMENTARY INFORMATIONS

Cuticular Hydrocarbon Composition of Adhesive Secretions from Functionally Different Attachment Pads of the Stick Insect *Medauroidea extradentata* (Phasmatodea)

JULIAN THOMAS*[1], STANISLAV N. GORB[1], THOMAS SCHMITT[2], THIES H. BÜSCHER[1†] and ZSOLT KÁRPÁTI[2,3†]

[1]*Functional Morphology and Biomechanics, Institute of Zoology, Kiel University, Am Botanischen Garten 1-9, 24118 Kiel, Germany*

[2]*Department of Animal Ecology and Tropical Biology Biocentre, University of Würzburg, Am Hubland, 97074 Würzburg, Germany*

[3]*Department of Chemical Ecology, HUN-REN, Centre for Agricultural Research, Plant Protection Institute, Fehérvári street 132-144, 1116, Budapest, Hungary*

**correspondence: Julian Thomas, jthomas@zoologie.uni-kiel.de*

[†]contributed equally to this work

E-mails: jthomas@zoologie.uni-kiel.de, tbuescher@zoologie.uni-kiel.de, sgorb@zoologie.uni-kiel.de, thomas.schmitt@uni-wuerzburg.de, zsolt.karpati@uni-wuerzburg.de*

Table S1: The hydrocarbon table containing our CHC profiles and all the CHC profiles we extracted from the previously published studies. The data can be downloaded via the following link: https://figshare.com/s/68aaa9f3e4dc0dcf7570.

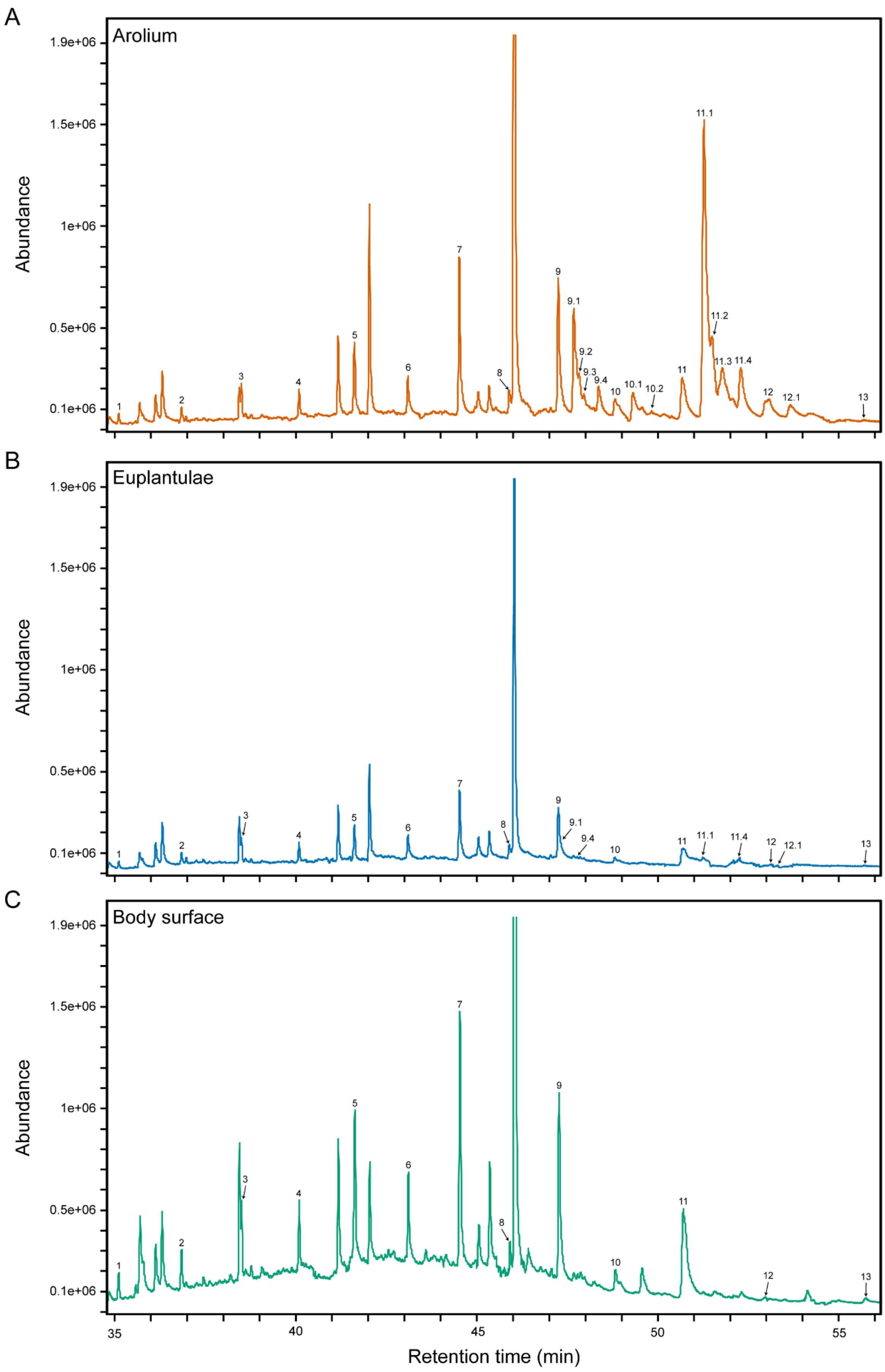
A
Arolium
B
Euplantulae
C
Body surface
Abundance
1.9e+06
1.5e+06
1e+06
0.5e+06
0.1e+06
1
2
3
4
5
6
7
8
9
9.1
9.2
9.3
9.4
10
10.1
10.2
11
11.1
11.2
11.3
11.4
12
12.1
13
35
40
45
50
55
Retention time (min)

Figure S1: Representative chromatograms of the three body parts. (A) Arolium, (B) Euplantulae, (C) Body surface. Each numbered peak represents one compound. 1 = C21, 2 = C22, 3 = C23, 4 = C24, 5 = C25, 6 = C26, 7 = C27, 8 = C28, 9 = C29, 9.1 = 15-; 13-MeC29, 9.2 = 7-MeC29, 9.3 = 5-MeC29, 9.4 = 3-MeC29, 10 = C30, 10.1 = 15-; 14-MeC30, 10.2 = 4-MeC30, 11 = C31, 11.1 = 15-; 13-MeC31, 11.2 = 7-MeC31, 11.3 = 5-MeC31, 11.4 = 3-MeC31, 12 = C32, 12.1 = 14-; 13-; 12-MeC32 13 = C33.

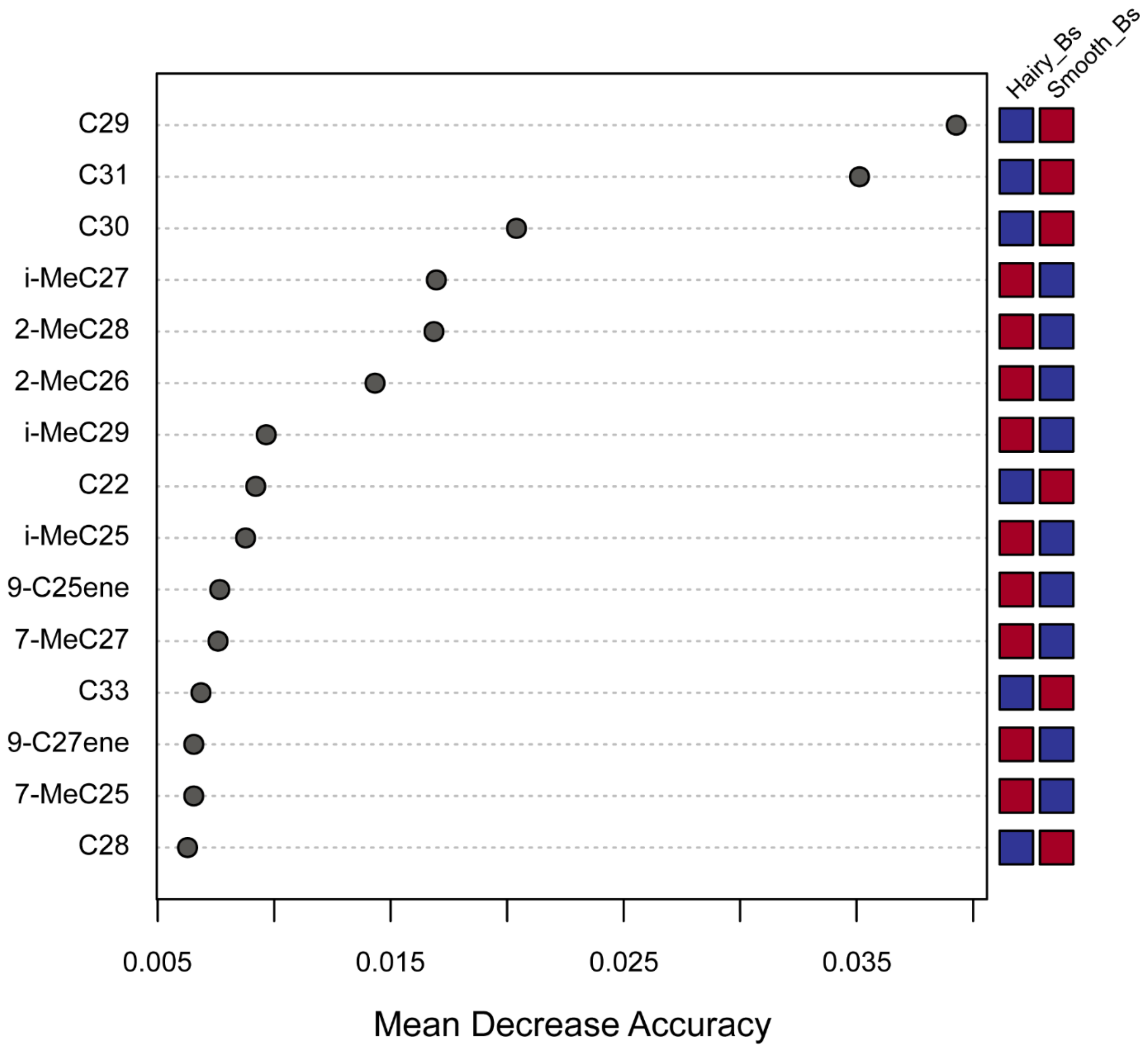

Figure. S2: Random Forest analyses of the hydrocarbon profiles from the body surface of insects with hairy and smooth attachment systems. Hydrocarbon profiles of the body surface from the insects with hairy (Hairy_Bs) and the smooth attachment systems (Smooth_Bs). On the y-axis are the 15 most influential hydrocarbons ranked by their mean decrease accuracy (mda). A higher mda value has a larger contribution to group separation. The squares on the right display the abundance of the corresponding hydrocarbons, with blue indicating low and red high abundance.

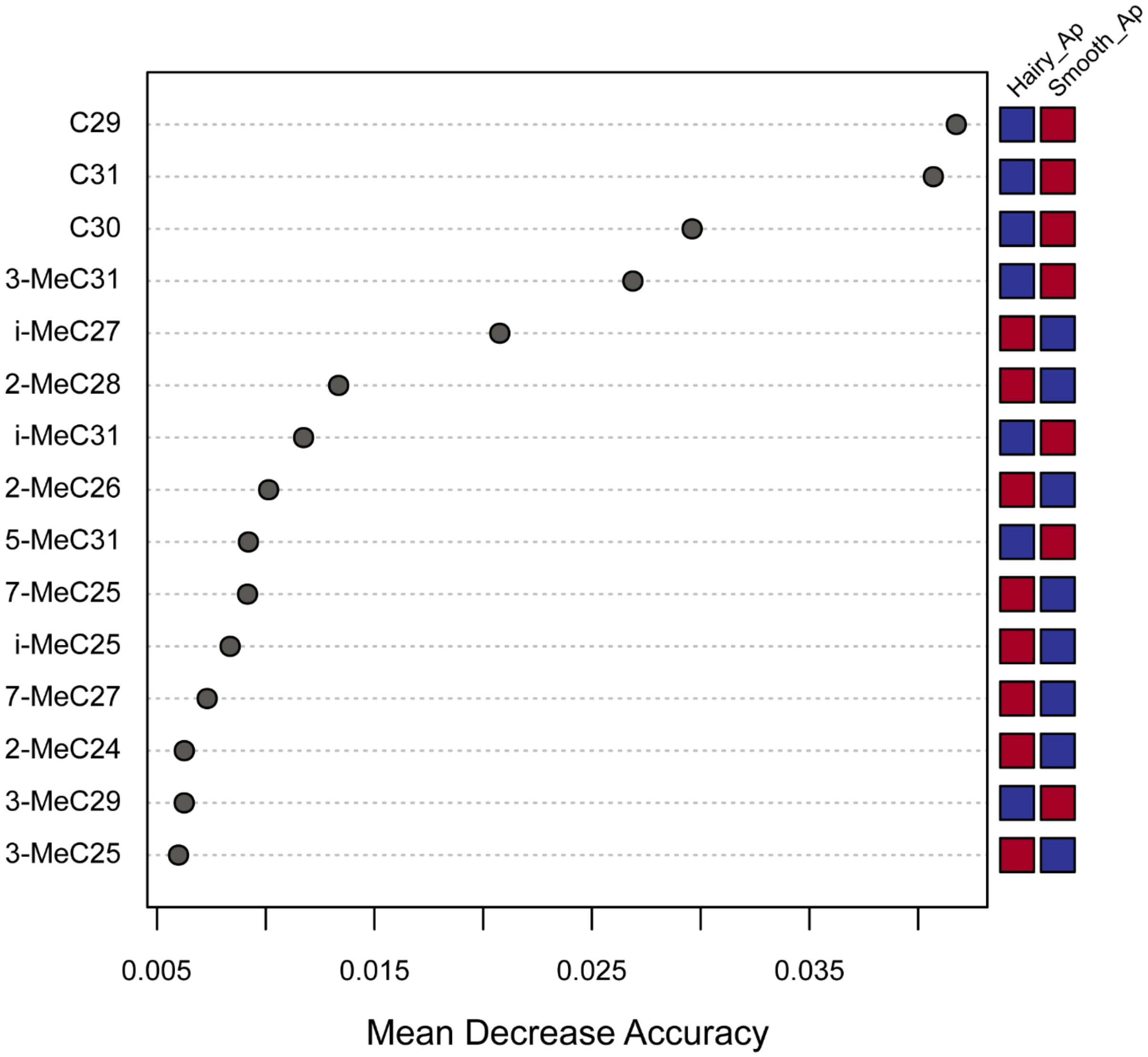

Figure. S3: Random Forest analyses of the hydrocarbon profiles from the attachment pads of hairy and smooth attachment systems. Hydrocarbon profiles of the attachment pads of hairy (Hairy_Ap) and smooth attachment systems (Smooth_Ap). On the y-axis are the 15 most influential hydrocarbons ranked by their mean decrease accuracy (mda). A higher mda value has a larger contribution to group separation. The squares on the right display the abundance of the corresponding hydrocarbons, with blue indicating low and red high abundance.

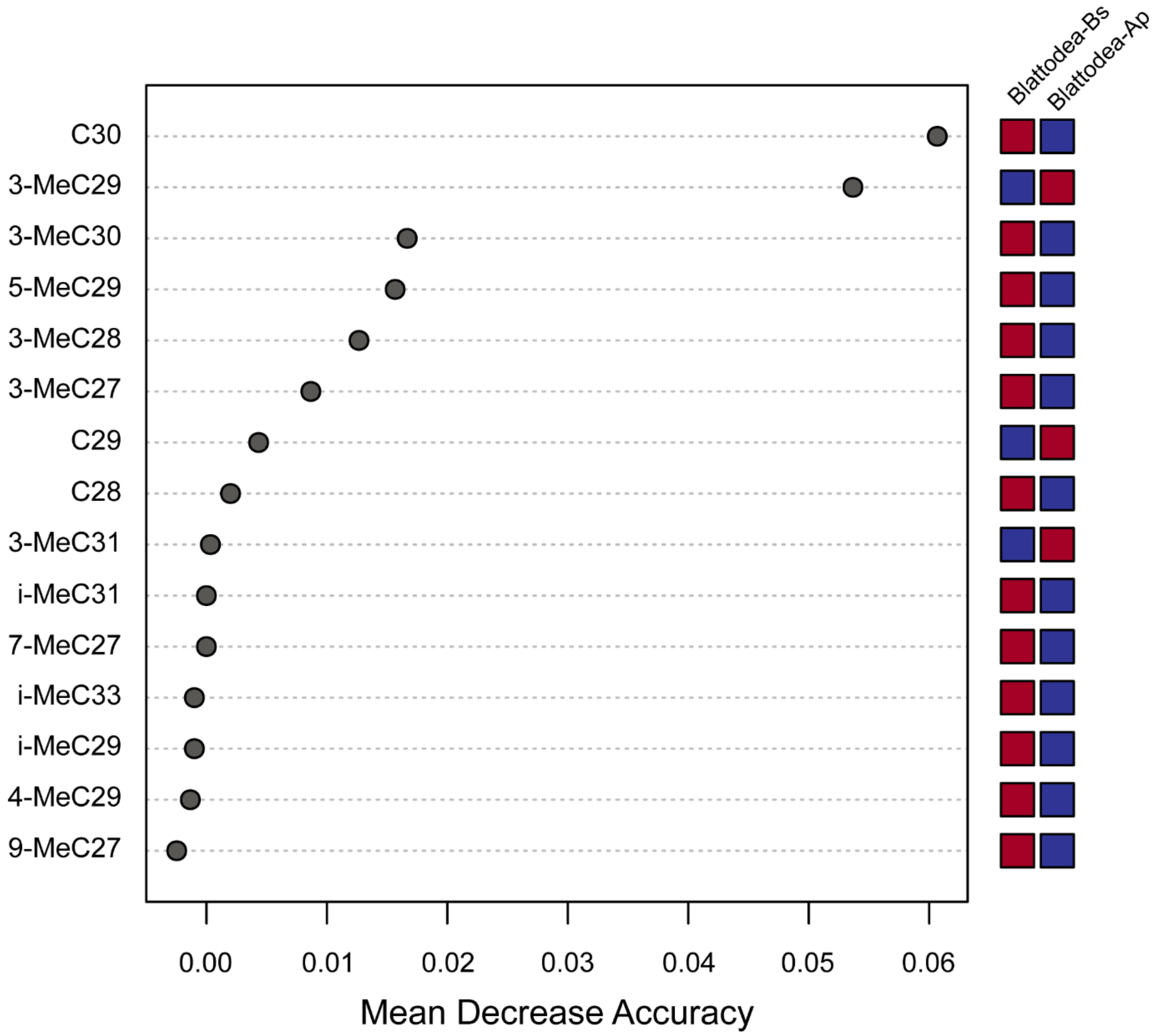

Figure. S4: Random Forest analyses of the hydrocarbon profiles from the attachment pads and body surface of Blattodea. Hydrocarbon profiles from the body surface of Blattodea (Blattodea-Bs) and from the attachment pads of Blattodea (Blattodea-Ap). On the y-axis are the 15 most influential hydrocarbons ranked by their mean decrease accuracy (mda). A higher mda value has a larger contribution to group separation. The squares on the right display the abundance of the corresponding hydrocarbons, with blue indicating low and red high abundance.
